# Evaluation of artificial intelligence-powered screening for sexually transmitted infections-related skin lesions using clinical images and metadata

**DOI:** 10.1186/s12916-024-03512-x

**Published:** 2024-07-18

**Authors:** Nyi N. Soe, Zhen Yu, Phyu M. Latt, David Lee, Jason J. Ong, Zongyuan Ge, Christopher K. Fairley, Lei Zhang

**Affiliations:** 1 Melbourne Sexual Health Centre, Alfred Health, 580 Swanston Street, Carlton, Melbourne, VIC 3053 Australia; 2 School of Translational Medicine, Faculty of Medicine, Nursing and Health Sciences, Monash University, Melbourne, Australia; 3 Augmented Intelligence and Multimodal analytics (AIM) for Health Lab, Faculty of Information Technology, Monash University, Melbourne, Australia; 4 Clinical Medical Research Centre, Children’s Hospital of Nanjing Medical University, Nanjing, Jiangsu Province 210008 China

**Keywords:** Artificial intelligence, Sexually transmitted infections, Clinical image classification

## Abstract

**Background:**

Sexually transmitted infections (STIs) pose a significant global public health challenge. Early diagnosis and treatment reduce STI transmission, but rely on recognising symptoms and care-seeking behaviour of the individual. Digital health software that distinguishes STI skin conditions could improve health-seeking behaviour. We developed and evaluated a deep learning model to differentiate STIs from non-STIs based on clinical images and symptoms.

**Methods:**

We used 4913 clinical images of genital lesions and metadata from the Melbourne Sexual Health Centre collected during 2010–2023. We developed two binary classification models to distinguish STIs from non-STIs: (1) a convolutional neural network (CNN) using images only and (2) an integrated model combining both CNN and fully connected neural network (FCN) using images and metadata. We evaluated the model performance by the area under the ROC curve (AUC) and assessed metadata contributions to the Image-only model.

**Results:**

Our study included 1583 STI and 3330 non-STI images. Common STI diagnoses were syphilis (34.6%), genital warts (24.5%) and herpes (19.4%), while most non-STIs (80.3%) were conditions such as dermatitis, lichen sclerosis and balanitis. In both STI and non-STI groups, the most frequently observed groups were 25–34 years (48.6% and 38.2%, respectively) and heterosexual males (60.3% and 45.9%, respectively). The Image-only model showed a reasonable performance with an AUC of 0.859 (SD 0.013). The Image + Metadata model achieved a significantly higher AUC of 0.893 (SD 0.018) compared to the Image-only model (*p* < 0.01). Out of 21 metadata, the integration of demographic and dermatological metadata led to the most significant improvement in model performance, increasing AUC by 6.7% compared to the baseline Image-only model.

**Conclusions:**

The Image + Metadata model outperformed the Image-only model in distinguishing STIs from other skin conditions. Using it as a screening tool in a clinical setting may require further development and evaluation with larger datasets.

**Supplementary Information:**

The online version contains supplementary material available at 10.1186/s12916-024-03512-x.

## Introduction

Sexually transmitted infections (STIs) pose a major public health challenge, with approximately one million new cases occurring daily worldwide [1, 2]. Australia is experiencing increasing rates of common STIs, including chlamydia, gonorrhoea and syphilis in recent years [3]. STIs have significant implications for individual health, including an increased risk of HIV acquisition, infertility, pregnancy complications and adverse birth outcomes. The resurgence of congenital syphilis, a severe consequence of untreated syphilis in pregnant women, has been observed in Australia, with 77 confirmed cases reported between 2016 and 2023 [4]. STIs also impose substantial economic burdens. In the United States, the direct lifetime medical costs associated with STIs were estimated at nearly $16 billion in 2018 [5].

Early diagnosis and treatment are critical to reduce the transmission of STIs and are key to effective STI control. The presence and nature of symptoms impact the effectiveness of STI control as infected individuals with noticeable symptoms will seek health care and treatment earlier compared to those without symptoms [6]. Even among symptomatic individuals, their health literacy and ability to recognise symptoms that are likely to be an STI also influence their health-seeking behaviours [7]. Healthcare providers have developed digital tools, including algorithms and symptom checker websites to promote early care-seeking [8–11]. Many machine learning approaches have been developed and evaluated for HIV and STI public health intervention, including risk assessment tools, symptom checkers and classifiers for certain anogenital conditions. Bao et al. [12] developed machine learning algorithms using demographic and sexual behaviour data to predict HIV and STI risk among men who have sex with men (MSM), with promising results. Xu et al. [10, 13, 14] advanced these algorithms for predicting the current risk and the future risk of acquiring these infections within 12 months. For symptomatic individuals, Soe et al.’s study [15] showed that the CatBoost model performed well in differentiating STIs from non-STI conditions, highlighting the potential of using deep learning algorithms to classify the anogenital skin conditions. However, there is no deep learning model specifically designed to classify skin conditions associated with STIs based on clinical images and presenting symptoms in Australia.

Recently, the use of artificial intelligence (AI) tools, particularly deep learning techniques such as convolutional neural networks (CNN), has been introduced in the healthcare sector. The AI approaches have shown promising results in assisting screening and diagnosis [16–18] and demonstrated good cost-effectiveness in implementation [19–21]. For example, studies have demonstrated that CNN can accurately distinguish between different skin lesions from dermoscopic and clinical images [22–26]. Brinker et al. [22] trained a CNN model on open-source dermoscopic images to classify melanoma images, demonstrating the potential of using such algorithms to assist dermatologists with melanoma detection. Hosny et al. [26] applied a refined residual deep convolutional network (RDCNN) to classify different skin lesions and achieved high accuracy on six skin cancer image datasets. Gonzalez-Alday et al. [27] demonstrated that CNN could reasonably classify images of herpes, warts and condylomas using a small dataset (*n* = 261) of genital lesions, achieving an accuracy of 86.6%. Alsahafi et al. [28] proposed an RDCNN to address the issue of an imbalanced dataset and demonstrated high accuracy for the multiclass classification of skin lesions. Hosny et al. [29] used a deep inherent learning approach to classify seven skin conditions from the HAM10000 dataset and applied explainable AI (X-AI) to assist the clinician with model interpretation. Other studies also showed the high accuracy of CNN models in identifying mpox skin lesions from other skin lesion images, with an area under the receiver operating characteristic curve (AUC) score exceeding 90% [30–32]. Additionally, recent studies showed the added value of integrating clinical metadata to improve the CNN model’s performance. For example, studies by Heo et al. and Ningrum et al. showed that integrating clinical metadata into CNN models enhanced accuracy in tuberculosis detection and melanoma classification [33, 34]. Liu et al. also demonstrated the potential of a multimodal approach to differentiate 26 skin conditions by integrating images and clinical data [24]. However, most existing studies have focused on general skin conditions, with only one study exploring the classification of anogenital skin lesions related to STIs, using a relatively small dataset. Only a few studies explored the integration of clinical metadata with images for improving model performance, but not specifically in the context of STI-related skin conditions. It highlights the need to evaluate the potential of a multimodal approach for distinguishing STIs from other skin conditions using larger and more diverse datasets.

This study aims to develop and evaluate a CNN model using clinical images to correctly determine if a lesion is an STI or not. In addition, we also aim to determine whether integrating epidemiological and clinical features into images improves the model performance in differentiating STIs from other skin conditions.

## Methods

We conducted this study at the Melbourne Sexual Health Centre (MSHC), which is the largest sexual health centre in Australia. We followed the MINimum Information for Medical AI Reporting (MINIMAR) recommendations for reporting study population, patient demographic characteristics, detailed information on model development and model evaluation [35].

### Data sources and collection

In this study, we used the clinical images and their corresponding patients’ information (metadata) acquired retrospectively from the files of 1648 MSHC clients. Informed consent was obtained from clients during the process of collecting images. The image dataset consisted of 4971 clinical images of skin lesions collected from 1 Jan 2010 to 23 Jan 2023. The images were taken using a compact digital camera or mobile phone camera by clinicians. The images contained (1) STI-related dermatological conditions (genital warts, herpes simplex virus, molluscum contagiosum, mpox, syphilis and syphilis rash) and (2) non-STI dermatological conditions (pearly penile papules, balanitis, dermatosis, lichen sclerosis, non-syphilis related skin rashes and healthy skin). Two medical students (DT, CK) manually extracted 21 metadata, including demographic information, presenting symptoms and final diagnosis from the clinical notes for each corresponding image (Additional file 1: Table S1). Two researchers (NS, PL) randomly selected and cross-checked 20% of the dataset at the start and 10% periodically throughout the data extraction process for accuracy and consistency. During cross-checks, any discrepancies in data extraction were discussed between the medical students and researchers to determine the final decision.

### Image selection criteria

Two experienced sexual health clinicians (CF, DL) and two researchers (NS, PL) checked the diagnosis of each image by reviewing the associated clinical notes and laboratory results from the clinic’s electronic health record (EHR)—Clinical Patient Management System (CPMS). Images without diagnostic consensus among the reviewers were excluded from our image dataset. We also checked all images to ensure they had no identifiable information such as faces, tattoos or birthmarks. We excluded 24 duplicate images and 16 low-resolution images from the image dataset. We also excluded 18 images as their corresponding metadata could not be identified in the (EHR) system.

### Data splitting

The final dataset contained 4913 de-identified images with corresponding metadata (1583 STIs and 3330 non-STIs). To reduce potential bias, we implemented a stratified fivefold cross-validation protocol in which the dataset was split into training (80%) and testing (20%) datasets. We grouped the images by the patient’s unique identifiers before splitting them to ensure that similar images taken from the same patients were not split between the training and testing datasets. This stratified splitting process was repeated five times, randomly shuffling the dataset before each iteration to generate varied allocations of data into each fold (Additional file 1: Fig. S1 and Table S2 for fivefold split details). The training dataset was used for training and internal validation, while the testing dataset was used as a hold-out dataset for external validation.

### Data pre-processing

We performed data pre-processing steps to prepare the image and metadata inputs for model training. For image data, we manually cropped each image to focus on the lesion areas and removed any distracting background content. This step ensured that the model’s attention was directed towards the relevant regions of interest. All cropped images were then resized to a standard dimension of 320 × 320 pixels to maintain consistent input sizes for the model. To achieve greater variation in the training dataset, we implemented data augmentation techniques. These included random cropping (extracting different sub-regions from the image), horizontal/vertical flips (creating mirrored versions of the image) and random adjustments to brightness and contrast levels. These augmentation techniques during model training improve the model’s generalisability and reduce overfitting. The metadata corresponding to each image consisted of both categorical and numerical variables. For the pre-processing of metadata, we used one-hot encoding for the categorical variables (e.g. gender, lesion site, etc.) and normalised the numerical variables (e.g. age and duration of lesion) to fall between 0 and 1.

### Model training

**Fig. 1:**
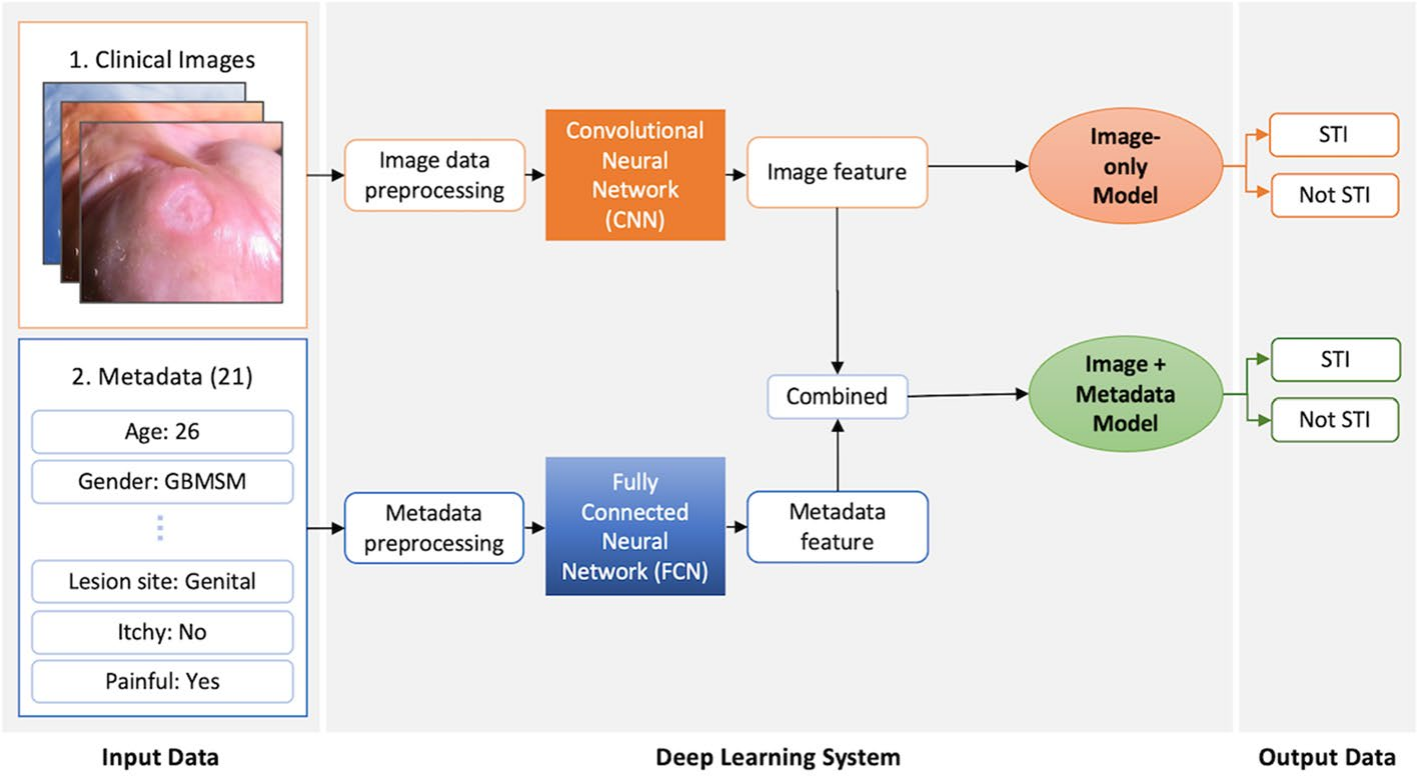
Schematic illustrating Image-only and Image + Metadata models. The detailed list of metadata can be seen in Table S1

We developed two binary classification models: a convolutional neural network (CNN) using images only and an integrated model (CNN + fully connected neural network (FCN)) using both images and metadata. For the CNN architecture, we employed a transfer learning approach, using a *Swin-Transformer* model pre-trained on a large image dataset [36, 37]. We fine-tuned this pre-trained model on our own image dataset to predict between STIs and non-STIs. Figure 1 shows an overview of Image-only and Image + Metadata models. In the Image-only model, the pre-processed images were passed through convolutional layers, which extracted image features. These image features were then input into classifier layers to generate predictions based only on images. In the Image + Metadata model, the image features extracted by the CNN were combined with the metadata features extracted by the FCN. These combined multimodal representations were then input into classifier layers to generate predictions based on both modalities. We implemented model training with *PyTorch* on a Tesla T4 GPU machine using Python programming language (version 3.8.2).

### Model evaluation

We assessed the model performance on the testing dataset, using the area under the receiver operating characteristic curve (AUC) as an evaluation metric (see Tables S3 and S4 for details). AUC measures the model’s ability to distinguish between two classes (STIs vs non-STIs), ranging from 0 to 1, where 1 reflects perfect classification [38, 39]. We also generated the receiver operating characteristic curve (ROC) by plotting the true positive rate (TPR) against the false positive rate (FPR) with different probability thresholds. The TPR and FPR are calculated as:

$$\mathrm{TPR}=\mathrm{True}\;\mathrm{Positives}/\left(\mathrm{True}\;\mathrm{Positives}+\mathrm{False}\;\mathrm{Negatives}\right)$$

$$\mathrm{FPR}=\mathrm{False}\;\mathrm{Positives}/\left(\mathrm{False}\;\mathrm{Positives}+\mathrm{True}\;\mathrm{Negatives}\right)$$

Then, we selected the optimal threshold to calculate sensitivity, specificity and accuracy. We performed five repeats of fivefold cross-validation, calculating the metrics for each fold in each repeat. We then calculated the mean and standard deviation (SD) of these metrics across the five repeats to produce the final reported performance measures.

### *Performance comparison between image-only and Image + Metadata models*

We utilised a paired *t*-test to examine the performance differences (AUC scores) between Image-only and Image + Metadata models on the same testing dataset folds. This assessed the statistical significance of the difference in AUC scores between the two models—one trained and evaluated on images only and the other on images along with associated metadata.

## Results

### Sample characteristics

Our study included 1583 STI and 3330 non-STI lesion images. Among STI lesion images, the most common diagnoses were syphilis (34.6%), genital warts (24.5%) and herpes simplex virus (19.4%). Among the non-STI images, the majority (80.3%) were genital skin conditions such as dermatitis, lichen sclerosis, balanitis and skin rashes, as shown in Table 1. In both STI and non-STI groups, the most frequently observed group was 25–34 years (48.6% and 38.2%, respectively) and heterosexual males (60.3% and 45.9%, respectively). For STI images, the most common anatomical locations were the male genitalia (52.0%) and anal/perianal regions (21.2%). For non-STI images, the male genitalia (45.0%) were also the most common locations, followed by female genitalia (20.5%). The most frequently observed duration of the lesion among STI images was 15–30 days (35.8%) and 8–14 days (25.5%), compared to 15–30 days (43.2%) and over 30 days (18.6%) for the non-STI images. Statistically significant differences (*p* values < 0.01) were observed in the distribution of age, gender, body region and lesion duration between STI and non-STI image groups.

**Table 1:** Distribution of images and corresponding metadata

	**STIs (column%)**	**Non-STIs (column%)**	*p* value**
**Number of images**			
Included (row%)	1583 (32.2%)	3330 (67.8%)	–
**Diagnoses**			
Genital warts	388 (24.5%)	0 (0.0%)	–
Herpes simplex virus	307 (19.4%)	0 (0.0%)	
Molluscum contagiosum	32 (2.0%)	0 (0.0%)	
Mpox	120 (7.6%)	0 (0.0%)	
Primary and secondary syphilis	547 (34.6%)	0 (0.0%)	
Rash of secondary syphilis	189 (11.9%)	0 (0.0%)	
Healthy skin (control)	0 (0.0%)	628 (18.9%)	
Pearly penile papules	0 (0.0%)	27 (0.8%)	
Other genital skin conditions*	0 (0.0%)	2675 (80.3%)	
**Age**			
18–24 years	417 (26.3%)	503 (15.1%)	< 0.01
25–34 years	770 (48.6%)	1273 (38.2%)	
35–44 years	274 (17.3%)	442 (13.3%)	
≥ 45 years	122 (7.7%)	484 (14.5%)	
Unknown	0 (0.0%)	628 (18.9%)	
**Gender**			
Heterosexual male	955 (60.3%)	1527 (45.9%)	< 0.01
Female	129 (8.1%)	1098 (33.0%)	
GBMSM	461 (29.1%)	598 (18.0%)	
Unknown	38 (2.4%)	107 (3.2%)	
**Lesions (number)**			
Single	377 (23.8%)	1393 (41.8%)	< 0.01
Multiple	1206 (76.2%)	1178 (35.4%)	
No	0 (0.0%)	759 (22.8%)	
**Region of body**			
Anal and perianal	336 (21.2%)	388 (11.7%)	< 0.01
Female genitalia	122 (7.7%)	681 (20.5%)	
Groin and pubis	18 (1.1%)	60 (1.8%)	
Head and neck	42 (2.7%)	191 (5.7%)	
Male genitalia	823 (52.0%)	1498 (45.0%)	
Torso	211 (13.3%)	178 (5.3%)	
Upper and lower extremities	23 (1.5%)	159 (4.8%)	
Unknown	8 (0.5%)	175 (5.3%)	
**Duration of presence**			
1–3 days	170 (10.7%)	146 (4.4%)	< 0.01
4–7 days	206 (13.0%)	161 (4.8%)	
8–14 days	403 (25.5%)	166 (5.0%)	
15–30 days	566 (35.8%)	1439 (43.2%)	
> 30 days	219 (13.8%)	618 (18.6%)	
Not applicable	19 (1.2%)	800 (24.0%)	
**Associated with pain**			
Yes	688 (43.5%)	622 (18.7%)	< 0.01
No	895 (56.5%)	2708 (81.3%)	
**Associated with itchiness**			
Yes	325 (20.5%)	810 (24.3%)	< 0.01
No	1258 (79.5%)	2520 (75.7%)	
**Associated with prodromal symptoms**			
Yes	257 (16.2%)	144 (4.3%)	< 0.01
No	1326 (83.8%)	3186 (95.7%)	

^*^Include balanitis, dermatitis, lichen sclerosis, lichen planus, pre-cancerous lesions, non-syphilis skin rashes

^**^The *p* values were derived from paired *t*-tests assessing the statistical significance in data distribution between STI and non-STI groups. A *p* value ≤ 0.05 indicates a statistically significant difference between the groups

### Model training findings

**Fig. 2:**
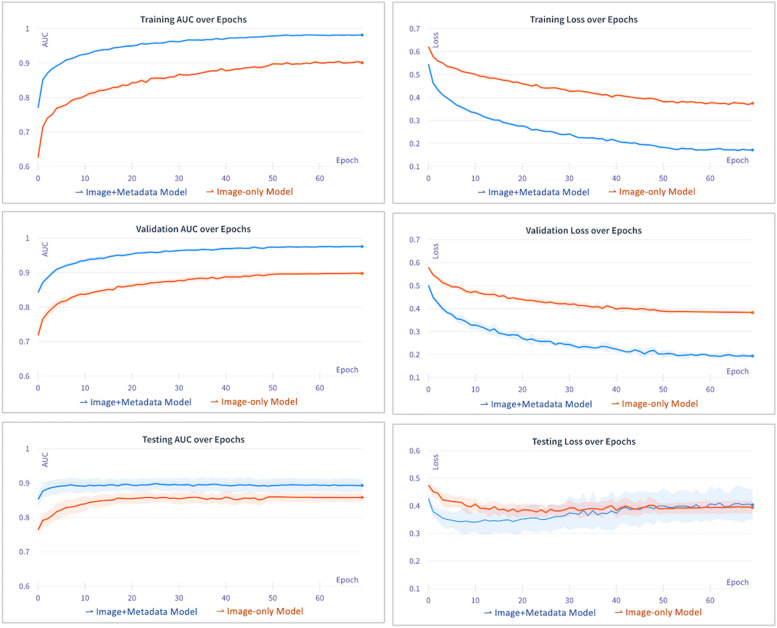
Model performance evaluation over training epochs for Image-only model and Image + Metadata model. Higher area under the receiver operating characteristic curve (AUC) indicates better discrimination between STIs and non-STIs and lower loss values indicates more effective training

During the model training process, we monitored the optimisation by tracking changes in AUC scores and loss values over each training round (epoch). A higher AUC score indicates better discrimination between STIs and non-STIs while a lower loss value indicates more learning by the model. The models were trained for 70 epochs, reaching a plateau where validation loss no longer declined. In the Image-only model, AUC climbed to around 0.900 during training and validation. Loss values declined over epochs, settling at 0.375 for training and 0.383 for validation. In comparison, the Image + Metadata model achieved higher AUC values around 0.970 during training and validation. Loss values were substantially lower at 0.172 for training and 0.194 for validation. The details are shown in Fig. 2 and Additional file 1: Table S2.

### Model evaluation findings

We evaluated the Image-only and Image + Metadata models on the testing dataset for each fold during fivefold cross-validations. We calculated the mean and standard deviation of the evaluation metrics for both models.

**Fig. 3:**
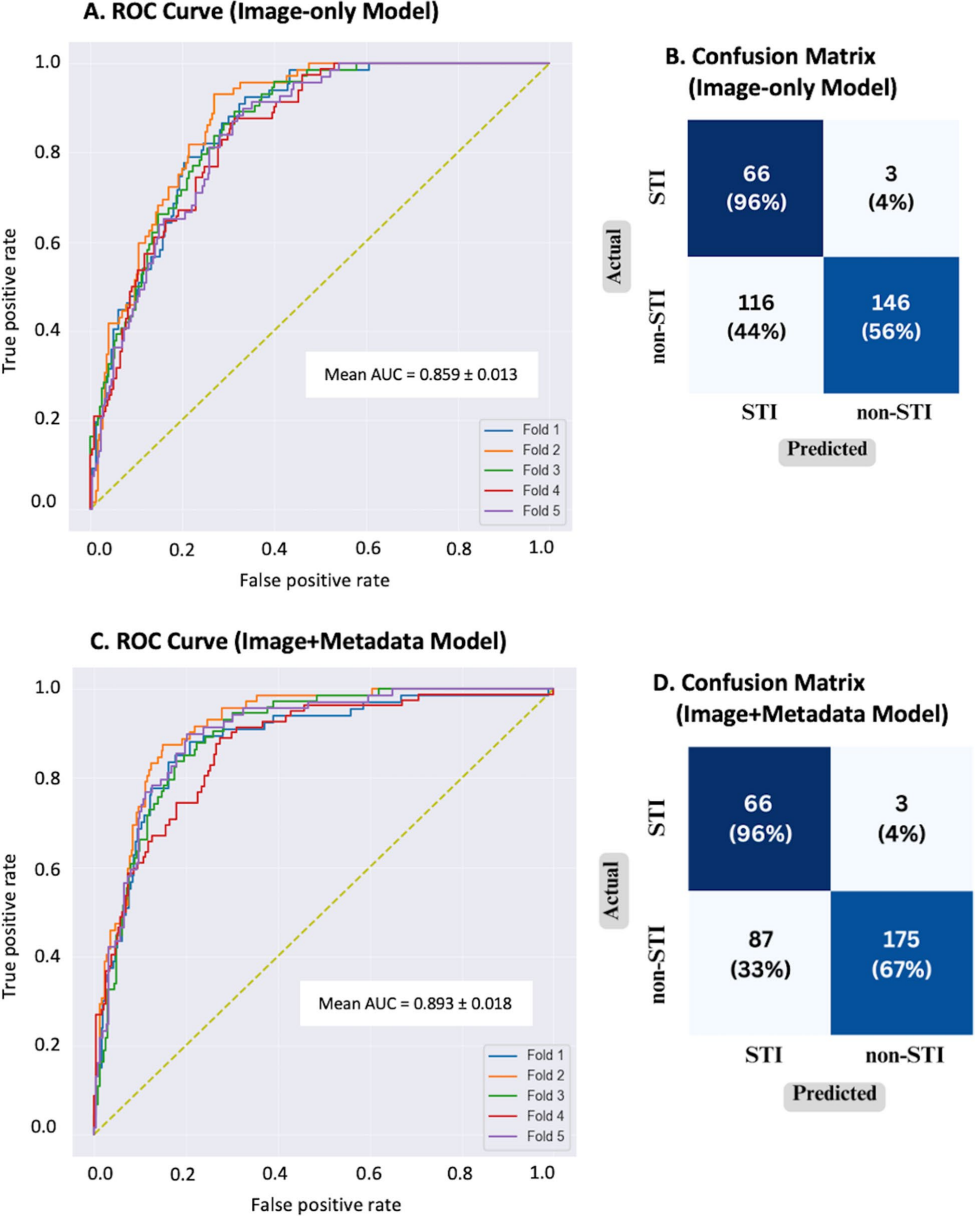
Performance of Image-only and Image + Metadata models on the testing cohort. **A** Receiver operating characteristic (ROC) curve of the CNN model showing true positive rate (TPR) vs false positive rate (FPR) across 5 cross-validation folds. **B** Confusion matrix for the Image-only model at fixed 95% sensitivity summarizing correct and incorrect predictions. **C** ROC curve of the Image + Metadata model showing TPR vs FPR across 5 cross-validation folds. **D** Confusion matrix for the Image + Metadata model at fixed 95% sensitivity indicating fewer misclassifications versus the Image-only model

On the testing dataset, the Image-only model achieved an AUC of 0.859 (SD 0.013), indicating its ability to reasonably distinguish between STIs and non-STIs. The optimal classification threshold was selected from ROC analysis (Fig. 3A) to optimise the sensitivity at 0.950. It achieved a sensitivity of 0.953 (SD 0.004), a specificity of 0.590 (SD 0.051), a precision of 0.359 (SD 0.040) and an accuracy of 0.669 (SD 0.043). The TPR and FPR were 0.957 and 0.443, respectively (a contingency table, Fig. 3B).

The Image + Metadata model achieved an AUC of 0.893 (SD 0.018), indicating higher distinguishing ability compared to the Image-only model. The ROC analysis curve is shown in Fig. 3C. At the optimal threshold, it achieved a sensitivity of 0.951 (SD 0.003), a specificity of 0.622 (SD 0.116), a precision of 0.433 (SD 0.061) and an accuracy of 0.692 (SD 0.093). The TPR and FPR were 0.957 and 0.324 respectively (Fig. 3D).

While comparing the Image-only and Image + Metadata models, both models achieved reasonable performance for differentiation between STI and non-STIs, where the Image + Metadata model outperformed the Image-only model. The Image + Metadata model achieved significantly higher AUC compared to the Image-only model (paired *t*-test, *p* < 0.01). The inclusion of metadata in the integrated model resulted in a relative improvement of 4% in the AUC compared to the Image-only model.

### Contribution of metadata on models’ performance

To better understand the contributory value of different metadata, we conducted the subgroup analyses by incrementally integrating metadata categories into the baseline Image-only model. The categories included three demographic, eight dermatological and ten genitourinary metadata (Additional file 1: Table S1). The inclusion of the 11 combined demographic and dermatological metadata led to the greatest improvement in model performance, increasing AUC by 6.71% compared to the baseline Image-only model. Individually, demographic and dermatological metadata contributed around 3–3.5% improvement in AUC. In contrast, including only genitourinary metadata did not contribute to performance improvement (details are shown in Table 2).

**Table 2:** Contribution of metadata on performance of Image-only model

Integration of metadata^a^	Number of features	AUC^b^	AUC improvement^c^ (%)
No metadata	0	0.846	–
Demographic	3	0.873	3.28
Dermatological	8	0.875	3.55
Genitourinary	10	0.845	− 0.04
Demographic + dermatological	11	0.902	6.71
Demographic + genitourinary	13	0.879	3.92
Dermatological + genitourinary	18	0.885	4.7
All metadata	21	0.901	6.51

^a^Table S1 provides details on specific metadata features in each category (demographic, dermatological and genitourinary)

^b^AUC = area under the receiver operation characteristic curve

^c^AUC improvement (%) were calculated compared to the AUC of the Image-only model

### Sensitivity–specificity trade-off across classification thresholds

Evaluation of the Image-only and Image + Metadata models across different classification thresholds showed a trade-off between sensitivity and specificity (Additional file 1: Table S5). Reducing sensitivity from 100 to 80% increased specificity from 45.8 to 74.0% for the Image-only model and 53.4 to 85.5% for the Image + Metadata model. Positive predictive value (PPV) also increased with higher specificity, rising from 32.7 to 44.7% (Image-only) and 35.4 to 58.7% (Image + Metadata). However, negative predictive value (NPV) declined slightly from 100 to 93.3% (Image-only) and 98.6 to 93.7% (Image + Metadata) at lower sensitivities. The analysis of false negative cases showed the specific types of STIs that were misclassified at different sensitivity levels. When the sensitivity was fixed at 95%, both the Image-only and Image + Metadata models misclassified one to two cases of syphilis and herpes as false negatives.

## Discussion

In this study, we demonstrate the first proof-of-concept showing that CNN models can feasibly distinguish STIs from other skin conditions in the clinical images, with and without additional patient metadata. The Image-only model showed a reasonable performance with an AUC of 0.859 in testing datasets. The integration of demographic and clinical metadata to images showed a significant improvement with a higher AUC of 0.893. When the sensitivity for detecting an STI is fixed at 95%, about one-third of non-STIs will be incorrectly classified but only two syphilis and one herpes out of 68 STIs will be incorrectly classified as a non-STI. Further research will be required to determine the potential acceptability and usefulness of such a service in clinical or public health settings, particularly in settings without access to STI diagnostics.

We compared our findings, specifically the discriminative ability measured by AUC, with other studies. Gonzalez-Alday et al. [27] used CNN to classify genital skin lesion images among herpes, warts and condyloma and achieved an accuracy of 0.866 but did not provide the AUC for direct comparison with our findings. Thieme et al. [30] demonstrated their CNN model can effectively distinguish mpox as a single disease from other skin conditions (mpox or non-mpox) with a very high AUC of 0.967. Our Image-only model achieved a lower AUC of 0.859 in distinguishing STIs from non-STI lesions. We included a more diverse range of common genital lesions in both STI and non-STI image groups than in previous studies, which may partially explain the lower AUC achieved by our model compared to disease-specific classification performance in previous studies. Distinguishing among these heterogenous lesions may be more challenging for our CNN model compared to differentiating mpox from other skin conditions.

In our study, the integration of 21 metadata, such as demographic information and symptoms, led to a 2.5–6.5% improvement in AUC compared to the baseline Image-only model. This aligns with the previous studies of skin lesion classification using clinical images and metadata. Liu et al. [24] showed that adding four demographic metadata to CNN models improved performance by 2.9% for the detection of tuberculosis using radiographic images, suggesting that the inclusion of more extensive metadata could improve the performance further. Liu et al. [24] used 45 metadata, including demographic information and medical history to integrate with the image model, improving performance by 4–5%. Our subgroup analysis (Table 2) showed that combined demographic and dermatological metadata contributed the most to achieving the greatest performance improvement. Genitourinary metadata did not contribute significantly to performance improvement and this may be because positive genitourinary findings rarely occurred (< 10% prevalence) together with skin conditions in our dataset.

While our model demonstrates potential as a screening tool, careful considerations and potential limitations must be addressed prior to considering its implementation in public health settings. First, maximizing sensitivity is essential for effective STI screening but has the problem that there are more false positives. In high-resource countries, where symptomatic patients are encouraged to get tested, the impact of overdiagnosis may be less significant [40]. Second, thoughtful interpretation and communication of the model’s predictions to the end-user are critical to avoid unintended consequences and promote health-seeking behaviour [41, 42]. In contrast to a sexual health clinic, where a thorough clinical history, examination and diagnostic testing are undertaken, our model could realistically just distinguish between a lesion that is likely to be an STI or one that is not. Therefore, our model’s prediction should be interpreted as indicating a “higher vs lower likelihood of an STI” rather than definitive “STI vs not STI” categorisations. Third, our data came from an STI clinic where the pretest probability of an STI was high because people had self-selected by being concerned they had an STI. If this was used by individuals who were not concerned about an STI but were labelled as possibly having one, it may have significant social and relationship consequences. As noted by Latt et al. [43], further research is needed to evaluate effective communication of the model’s predictions to the users to improve healthcare seeking while avoiding unnecessary concern, given the sensitive nature of sexual health. Fourth, the application of deep learning in the medical field has been controversial due to the black box phenomenon, where the interpretive mechanisms between input and output remain unexplained [34]. To address this, the interpretation of the model should be explained using visualisation techniques such as Grad-CAM or SHapley Additive exPlanations (SHAP) to facilitate a better understanding of the decision-making process for the end-user [44]. Finally, unlike other diseases, our focus on STI skin lesions raises privacy and security concerns due to the need to use images from private areas. Therefore, it is essential to assess the feasibility, acceptability and preference of its use prior to the application of the tool.

Our study provides a novel approach to addressing a research gap in the sexual health domain by demonstrating the feasibility of using a multimodal deep learning approach to distinguish STIs from other conditions. We used a larger dataset of anogenital skin lesion images, including a wide range of STI and non-STI lesions. Additionally, we applied fivefold cross-validation to evaluate the robustness of the performance of our models. However, our study has limitations. First, our study was based on retrospective data from a single sexual health clinic in Victoria, which may introduce a potential bias towards cases with more typical presentations or unusually severe cases. External validation with data from other clinics was not feasible in this initial study, however will be an important next step to validate the generalisability of the model to ensure the robustness and applicability of the findings. There was also a significant gender imbalance in the dataset, with females representing only 8.1% of STI cases. While we employed techniques such as data augmentation and stratified cross-validation to mitigate potential bias, this gender imbalance may have impacted the model’s predictive accuracy, particularly for females with STIs. Second, while differentiating STIs from non-STIs, there are still limitations in detecting important STIs such as syphilis, even with 95% sensitivity. More images and data are required to optimise the model’s performance, especially for syphilis. We only included a subset of the available images from our centre due to the intensive resources required for the manual extraction of metadata from EHR. However, the current dataset was sufficient for the proof-of-concept application of deep learning in STI lesion classification. Third, the scope of this study was limited to binary classification between STIs and non-STIs. Future research should address multiclass discrimination problems among diverse genital lesions to provide a comprehensive understanding of deep learning’s ability to differentiate different features of specific conditions. Finally, we used reduced 320 × 230 image resolutions due to the computational constraints, which may have affected the model’s performance. Furthermore, the images were captured from different sources, including digital cameras and mobile phone cameras, which could potentially introduce variation in image quality and resolution despite data pre-processing to standardise resolutions. Further studies should experiment with the impact of using higher resolution images to improve the model performance potentially.

## Conclusions

Our study demonstrated that CNNs could reasonably distinguish STIs from other anogenital conditions using clinical images. Integrating demographic and clinical metadata with images further improved accuracy. These findings open up avenues for further research into developing AI-assisted tools that could potentially assist public health measures for early detection and treatment of STs. To be useful as a screening tool, further development and evaluation studies with larger datasets should be conducted.

### Supplementary Information

Suppelementary Material 1.

## Data Availability

The dataset used in this study is not publicly available due to privacy and consent limitations. It consists of non-identifiable anogenital images and associated metadata provided by clients, who did not consent to have their data shared openly.

## Abbreviations

AI: Artificial intelligence
AUC: Area under the ROC curve
CNN: Convolutional neural network
CPMS: Clinical Patient Management System
EHR: Electronic health record
FCN: Fully connected neural network
FPR: False positive rate
MINIMAR: MINimum Information for Medical AI Reporting
MSHC: Melbourne Sexual Health Centre
NPV: Negative predictive value
PPV: Positive predictive value
RDCNN: Residual deep learning neural network
SHAP: SHapley Additive exPlanations
STIs: Sexually transmitted infections
TPR: True positive rate
X-AI: Explainable AI
