# Supplementary material for: Evaluation of artificial intelligence-powered screening for sexually transmitted infections-related skin lesions using clinical images and metadata
Source: BMC Med. 2024 Jul 18;22:296. doi: 10.1186/s12916-024-03512-x (PMC11256573; doi:10.1186/s12916-024-03512-x)
Supplement: Supplementary file 1 — Suppelementary Material 1. [file 12916_2024_3512_MOESM1_ESM.docx]

Table S1 Metadata extracted from the electronic health record system

| No | Categories | Variables/ Metadata | Definition | Drop-down list for options |
| --- | --- | --- | --- | --- |
| 1 | Demographic characteristics | Record Number | A unique identifier assigned to each record | free text (number) |
| 2 |  | Date | The date of the record entry | free text (date format) |
| 3 |  | De-identified Patient Number | Deidentified Unique Number assigned to each patient | free text |
| 4 |  | Age | Age of the client at the time of consultation in years | free text (number) |
| 5 |  | Gender | Gender of the client | GBMSM, Male, Female, Unknown |
| 6 |  | On PrEP | Indicates whether the patient is on pre-exposure prophylaxis (PrEP) medication at the time of consultation | Yes, No, Unknown |
| 7 | Dermatological symptoms/signs | Site | Anatomical location of the lesion | Genital, non-Genital, No |
| 8 |  | Lesion Number | Number of lesions observed | Single, Multiple, No |
| 9 |  | Lesion Duration (days) | Duration of lesion presence in days | free text (number) |
| 10 |  | Itchy | Presence of itching accompanying the lesion | Yes, No |
| 11 |  | Painful | Presence of pain accompanying the lesion | Yes, No |
| 12 |  | Prodromal Symptom | Presence of prodromal symptoms (e.g., fever, malaise, anorexia, headache) prior to the appearance of the lesion | Yes, No |
| 13 |  | Skin Disorder History | Presence of any skin disorders history | Yes, No |
| 14 |  | Allergic Reaction History | Presence of allergic reaction history | Yes, No |
| 15 | Genitourinary symptoms/signs | Dysuria | Presence of painful or burning urination | Yes, No |
| 16 |  | Abnormal Discharge | Presence of abnormal discharge from the penis or vagina | Yes, No |
| 17 |  | Discharge Colour | Colour of abnormal discharge | Clear/Whitish, Green, Grey/Brown, Yellow, No |
| 18 |  | Discharge Smell | Presence of noticeable odour in an abnormal discharge | Yes, No |
| 19 |  | Abnormal Vaginal Bleeding | Presence of abnormal vaginal bleeding | Yes, No |
| 20 |  | Pain During Sex | Presence of pain during sexual intercourse in female client | Yes, No |
| 21 |  | Testicular Pain | Presence of pain or discomfort in the testicles | Yes, No |
| 22 |  | Anorectal Pain | Presence of pain or discomfort from the anal area | Yes, No |
| 23 |  | Anorectal Bleeding | Presence of abnormal bleeding from the anal area | Yes, No |
| 24 |  | Abnormal Bowel Symptom | Presence of any associated abnormal bowel symptoms | Yes, No |
| 25 | Diagnosis | Diagnosis (STI/non-STI) | Classification of the final diagnosis as STI or non-STI | STI, non-STI |

Figure S1 5-folds cross-validation for data splitting into training and testing cohorts


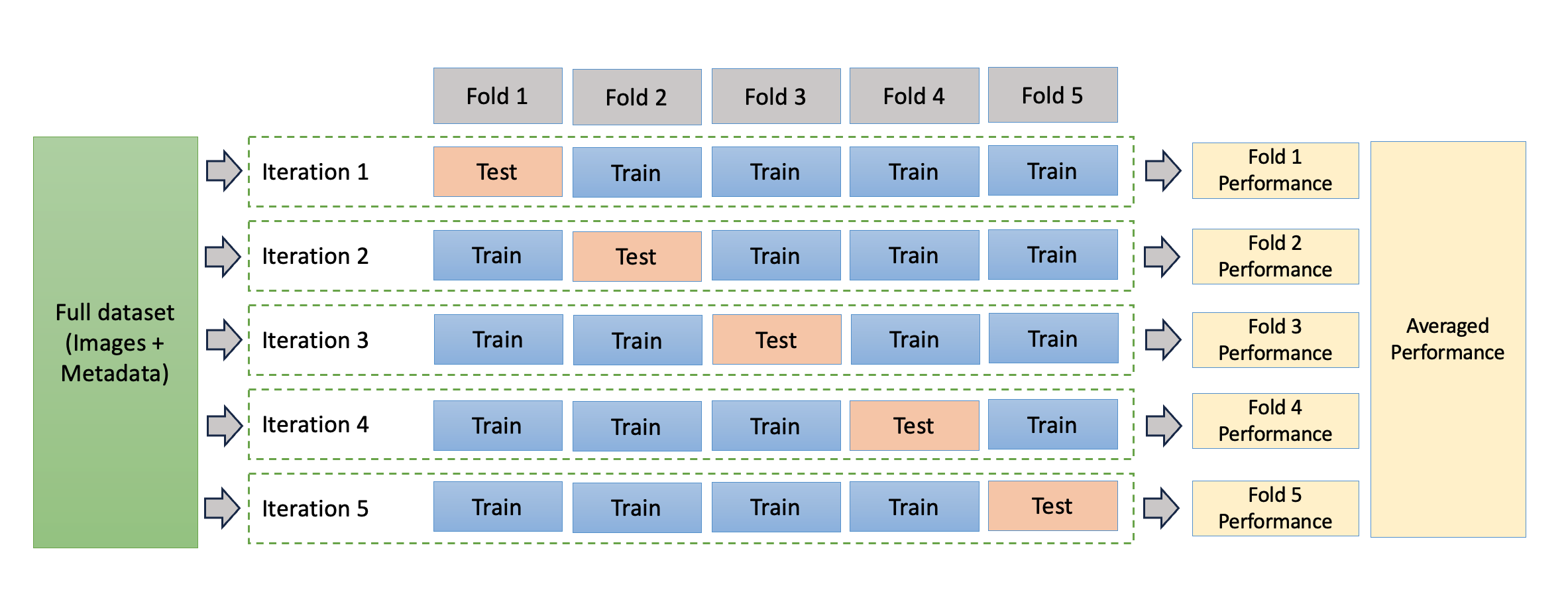


Table S2 Distribution of STI and non-STI images between the training and testing cohorts with 5-folds cross-validation

| **Data Splitting** | **Training Dataset (80%)** | | **Testing Dataset (20%)*** | |
| --- | --- | --- | --- | --- |
|  | **STIs** | **non-STIs** | **STIs** | **non-STIs** |
| **Fold 1** | 1,352 | 2,609 | 67 | 265 |
| **Fold 2** | 1,402 | 2,490 | 72 | 260 |
| **Fold 3** | 1,332 | 2,611 | 73 | 252 |
| **Fold 4** | 1,359 | 2,582 | 82 | 245 |
| **Fold 5** | 1,382 | 2,532 | 69 | 262 |

**Similar images from the same patient were excluded in the testing dataset after an 80-20 split*

Table S3 Confusion matrix calculation

| **Output** | | **Predicted Diagnosis** | |
| --- | --- | --- | --- |
|  |  | STI | non-STI |
| **Actual Diagnosis** | STI | TP | FN |
|  | non-STI | FP | TN |

TP: True Positive = Number of correctly classified STI cases

TN: True Negative = Number of correctly classified non-STI cases

FP: False Positive = Number of incorrectly classified STI cases

FN: False Negative = Number of incorrectly classified non-STI cases

Table S4 Performance evaluation metrics

| **Performance criteria** | **Calculation** |
| --- | --- |
| Accuracy | (TP + TN) / (TP + TN + FP + FN) |
| Sensitivity (recall) | TP / (TP + FN) |
| Specificity | TN/ (TN + FP) |
| TPR | TP / (TP + FN) |
| FPR | FP / (FP + TN) |
| FNR | FN / (FN + TP) |
| PPV (precision) | TP / (TP + FP) |
| NPV | TN / (TN + FN) |
| AUC | plots TPR against FPR at different thresholds |

TPR: true positive rate; FPR: false positive rate; FNR: false negative rate; PPV: positive predictive value; NPV: negative predictive value

Table S5 Models’ performance at different fixed sensitivity levels

| 1. **Image-Only Model** | | | | | |
| --- | --- | --- | --- | --- | --- |
| **Fixed Sensitivity at** | **Detect all Syphilis*** | **95%** | **90%** | **85%** | **80%** |
| Threshold | 0.022 | 0.066 | 0.171 | 0.240 | 0.269 |
| AUC | 0.846 | 0.846 | 0.846 | 0.846 | 0.846 |
| Sensitivity (recall) | 1.000 | 0.957 | 0.900 | 0.850 | 0.800 |
| specificity | **0.458** | **0.557** | **0.668** | **0.718** | **0.740** |
| accuracy | **0.571** | **0.637** | **0.713** | **0.740** | **0.752** |
| PPV (precision) | 0.327 | 0.359 | 0.412 | 0.435 | 0.447 |
| NPV | 1.000 | 0.973 | 0.956 | 0.940 | 0.933 |
| FNR (FN /FN+TP) | 0.000 | 0.692 | 0.742 | 0.767 | 0.779 |
| FPR (FP/FP+TN) | 0.542 | 0.443 | 0.332 | 0.282 | 0.260 |
| **Breakdown of False Negative Cases** | | | | | |
| Total FN Cases | 0 | 3 | 8 | 12 | 14 |
| Syphilis (n=15) | 0 | 1 | 2 | 2 | 3 |
| Syphilis-Rash (n=4) | 0 | 0 | 0 | 0 | 0 |
| Mpox (n=3) | 0 | 0 | 0 | 0 | 0 |
| Herpes (n=15) | 0 | 1 | 3 | 3 | 3 |
| Warts (n=22) | 0 | 0 | 2 | 4 | 5 |
| Molluscum (n=2) | 0 | 0 | 0 | 1 | 1 |
| Other (n=270) | 0 | 1 | 1 | 2 | 2 |

** The optimal classification threshold was selected from the Receiver Operating Characteristic Curve (ROC) analysis to detect all syphilis cases.*

| 1. **Image+Metadata Model** | | | | | |
| --- | --- | --- | --- | --- | --- |
| **Fixed Sensitivity at** | **Detect all Syphilis*** | **95%** | **90%** | **85%** | **80%** |
| Threshold | 0.014 | 0.052 | 0.162 | 0.238 | 0.386 |
| AUC | 0.901 | 0.901 | 0.901 | 0.901 | 0.901 |
| Sensitivity (recall) | 0.971 | 0.957 | 0.900 | 0.850 | 0.800 |
| specificity | **0.534** | **0.676** | **0.798** | **0.821** | **0.855** |
| accuracy | **0.625** | **0.731** | **0.816** | **0.825** | **0.840** |
| PPV (precision) | 0.354 | 0.433 | 0.535 | 0.552 | 0.587 |
| NPV | 0.986 | 0.978 | 0.963 | 0.951 | 0.937 |
| FNR (FN /FN+TP) | 0.029 | 0.731 | 0.774 | 0.788 | 0.806 |
| FPR (FP/FP+TN) | 0.466 | 0.324 | 0.202 | 0.179 | 0.145 |
| **Breakdown of False Negative Cases** | | | | | |
| Total FN Cases | 2 | 4 | 8 | 10 | 14 |
| Syphilis (n=15) | 0 | 2 | 3 | 3 | 3 |
| Syphilis-Rash (n=4) | 0 | 0 | 0 | 0 | 0 |
| Mpox (n=3) | 0 | 0 | 0 | 0 | 1 |
| Herpes (n=15) | 1 | 1 | 3 | 3 | 4 |
| Warts (n=22) | 0 | 0 | 1 | 3 | 4 |
| Molluscum (n=2) | 0 | 0 | 0 | 0 | 0 |
| Other (n=270) | 1 | 1 | 1 | 1 | 2 |

** The optimal classification threshold was selected from the Receiver Operating Characteristic Curve (ROC) analysis to detect all syphilis cases.*

Figure S2 Models’ optimization and hardware utilization over training epochs
